# Sensory Memory Is Allocated Exclusively to the Current Event-Segment

**DOI:** 10.3389/fpsyg.2018.01435

**Published:** 2018-09-07

**Authors:** Srimant P. Tripathy, Haluk Öǧmen

**Affiliations:** ^1^School of Optometry and Vision Science, University of Bradford, Bradford, United Kingdom; ^2^Department of Electrical and Computer Engineering, University of Denver, Denver, CO, United States

**Keywords:** memory, sensory memory, iconic memory, short-term memory, modal model of memory, event segmentation, tracking, multiple-object tracking

## Abstract

The Atkinson-Shiffrin modal model forms the foundation of our understanding of human memory. It consists of three stores (Sensory Memory (SM), also called iconic memory, Short-Term Memory (STM), and Long-Term Memory (LTM)), each tuned to a different time-scale. Since its inception, the STM and LTM components of the modal model have undergone significant modifications, while SM has remained largely unchanged, representing a large capacity system funneling information into STM. In the laboratory, visual memory is usually tested by presenting a brief static stimulus and, after a delay, asking observers to report some aspect of the stimulus. However, under ecological viewing conditions, our visual system receives a continuous stream of inputs, which is segmented into distinct spatio-temporal segments, called events. Events are further segmented into event-segments. Here we show that SM is not an unspecific general funnel to STM but is allocated exclusively to the current event-segment. We used a Multiple-Object Tracking (MOT) paradigm in which observers were presented with disks moving in different directions, along bi-linear trajectories, i.e., linear trajectories, with a single deviation in direction at the mid-point of each trajectory. The synchronized deviation of all of the trajectories produced an event stimulus consisting of two event-segments. Observers reported the pre-deviation or the post-deviation directions of the trajectories. By analyzing observers' responses in partial- and full-report conditions, we investigated the involvement of SM for the two event-segments. The hallmarks of SM hold only for the current event segment. As the large capacity SM stores only items involved in the current event-segment, the need for event-tagging in SM is eliminated, speeding up processing in active vision. By characterizing how memory systems are interfaced with ecological events, this new model extends the Atkinson-Shiffrin model by specifying how events are stored in the first stage of multi-store memory systems.

## Introduction

**Figure 1:**
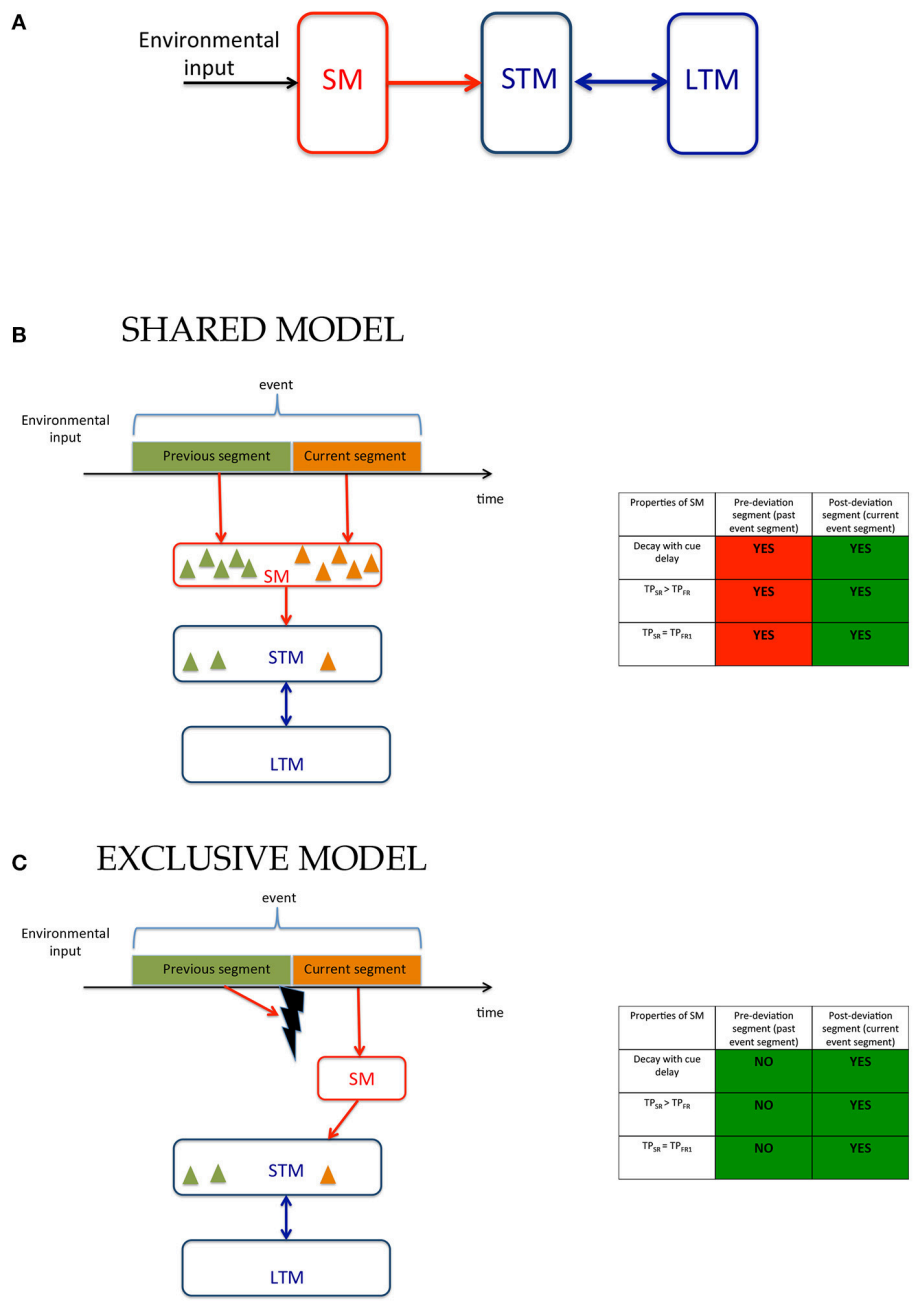
Models of human memory. **(A)** The modal model: The environmental input is received by SM whose contents are transferred to STM and LTM. **(B)** Working of the modal model in ecological viewing where the environmental input is a continuous stream of signals that are segmented into events containing sub-events, i.e., segments. The observer encodes in memory not only stimuli present within the event but for each stimulus a tag that indicates to which segment it belongs. The contents of the memory are depicted by triangles whose color indicates segment tags. **(C)** An alternative model where SM is exclusively allocated to the current segment of the event. When event segmentation is driven by low-level stimulus changes, transients may be used to mask and reset SM (Averbach and Coriell, 1961; Breitmeyer and Öǧmen, 2006) so that it can now be allocated to the current event segment. Additional mechanisms may be involved when event segmentation is based on higher-level criteria. The model in **(B)** has the advantage that the large capacity SM can be used for all segments of an event within the time-span of SM, while the model in **(C)** can use the capacity of SM only for the current event. However, the advantage of this model is that it requires less information to be stored in SM and requires much fewer tagging operations since the contents of the large capacity SM are exclusively allocated to the current event-segment and do not need tagging. The tables to the right of **(B,C)** show the predictions of these models and the experimental support for the predictions. The model in **(B)** involves SM for both pre- and post-deviation segments and thus, as indicated by “YES” entries, predicts that properties of SM should be found for both segments. The model in **(C)**, on the other hand predicts that properties of SM should be found only for the current event segment. Thus, the models differ in their predictions for the pre-deviation segment. The green and red colors in each box indicate experimental agreement and disagreement, respectively, with the predictions.

The classical multi-store (or modal) model of human memory (Atkinson and Shiffrin, 1968, 1971) posits that information is encoded and stored in three memory systems (Figure 1A): First, a large-capacity but rapidly decaying SM stores information for a few 100 ms. A subset of the contents of SM is transferred into a more durable STM, which can store items for a few seconds. However, STM's capacity is severely limited. Finally, some items in STM are transferred into LTM whose contents can last as long as a lifetime.

Since its inception, the STM and LTM components of the modal model have undergone significant modifications (Baddeley and Hitch, 1974; Klatzky, 1980; Cowan, 2001; Alvarez and Cavanagh, 2004; Baddeley, 2007; Bays and Husain, 2008; Zhang and Luck, 2008; Van den Berg et al., 2012), while the classical view of SM as a large capacity system funneling a static snapshot of visual inputs into STM has persisted for several decades (Sperling, 1960; Averbach and Coriell, 1961; Coltheart, 1983; Haber, 1983; Breitmeyer and Öǧmen, 2006). Recent models extended SM in terms of its architecture and function.

In terms of how SM is implemented in the brain, recently a multi-layer approach reflecting the hierarchy of the visual cortex has been proposed (Rensink, 2014). It was found that the retention-duration in SM depends on the nature (but not on the difficulty) of the task that SM is called to subserve (Rensink, 2014). For change detection, target-identity report, and static detection tasks, the corresponding SM retention-durations were 120, 190, and 240 ms, respectively. This finding was interpreted to reflect a multi-layer cortical implementation of SM, according to which SM can store information and make it available both within and across the cortical layers by relying on horizontal and vertical feedback, respectively. Hence, according to this model, the architecture of SM is distributed across the cortex following the hierarchy of visual cortical processes. This distributed implementation suggests that information flow from SM to STM and to other visual processes occurs within and across cortical layers because, architecturally, SM is an integral part of various visual processing networks.

Another aspect of cortical organization is retinotopy: Via the optics of the eyes, neighboring points in the environment are mapped to neighboring points on the retina and these neighborhood relations are preserved in early visual cortical areas. Under ecological viewing conditions, the observer (eyes, head, body) and many objects in the environment are in motion. While a retinotopically encoded SM can store and process information that is stable (i.e., static) with respect to its retinotopic reference-frame, the storage and processing of dynamic information is problematic (Haber, 1983): Any relative motion between the observer's retinae and the external environment will cause a shift in retinotopic coordinates for the stimulus received by SM. These shifts, in turn, will cause spatiotemporal blurring and inappropriate integration of information over space and time (Öǧmen, 2007; Öǧmen and Herzog, 2010). Based on a series of experiments that show non-retinotopic storage and processing of information, recently a modified SM model has been suggested (Öǧmen and Herzog, 2016). According to this model, SM has two parallel components, one based on a retinotopic reference-frame (*r*SM) and a second one based on motion-grouping based (non-retinotopic) reference frames (*nr*SM).

Another recent modification to the multi-store functional architecture of memory systems has been the introduction of a new visual memory component, intermediate between SM and STM (Sligte et al., 2008; van Moorselaar et al., 2015). This component is proposed to have large capacity and a retention time in the order of several seconds. It was suggested that this memory component lacks robustness with respect to changing inputs and hence was termed *fragile visual STM*. Evidence suggests that the erasure of the contents of the fragile STM depends on both location-specific and object-specific matches between the contents and the new input, leading to the proposal that this memory component holds higher object-level representations but that these representations are anchored to specific locations (Pinto et al., 2013). However, other studies have questioned the existence of fragile VSTM (Matsukura and Hollingworth, 2011; Makovski, 2012).

In the laboratory, visual memory is usually tested by presenting a brief static stimulus (e.g., shapes with different colors) and, after a delay, asking observers to report some aspect of the stimulus. However, under normal ecological viewing conditions, due to the movements of the observer and those of objects in the environment, our visual system receives a continuous stream of dynamic inputs. Perceptually, the visual system segments the continuous stream of visual information into distinct spatio-temporal segments, called events (Johansson et al., 1980; Warren and Shaw, 1985). For example, flipping a coin can be considered as an event, which in turn can have sub-segments (Zacks and Swallow, 2007), such as the launching of the coin, following the movement of the coin in the air, and catching the coin, all occurring within a second. An important question is then how events and their segments are stored in SM. Previous studies examined how information embedded in events is stored in STM and LTM. It has been reported that objects that were present around event boundaries were better recalled than other objects in the stimulus (Swallow et al., 2009). This finding has been interpreted as evidence that event boundaries structure the contents of STM and LTM (Swallow et al., 2009). Given this background, our goal was to analyse the relationship between event segments and memory mechanisms, in particular for relatively short events that span the first two stages of the modal model, namely SM and STM. We focused on these two memory stores because, due to their time-scales, they are inherently involved in real-time ecological aspects of perception and cognition and because they can be distinguished from each other by well-established experimental techniques (Sperling, 1960; Averbach and Coriell, 1961; Coltheart, 1983; Haber, 1983; Breitmeyer and Öǧmen, 2006).

**Figure 2:**
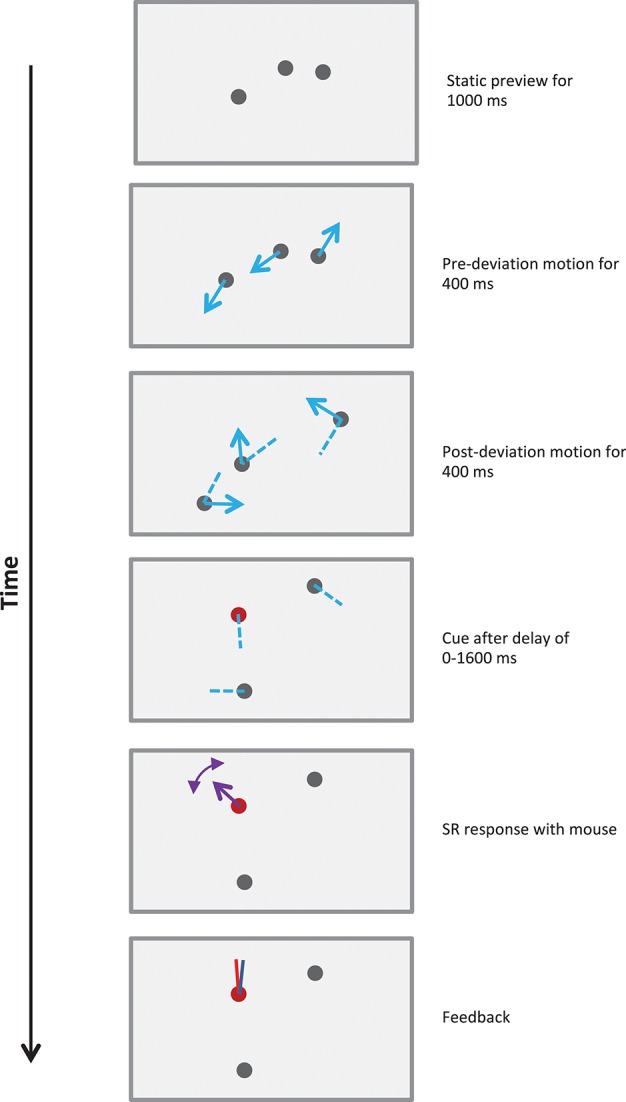
Schematic of a 800 ms stimulus in Experiment 1. Each trial started with three randomly positioned stationary disks presented on the screen. The observer indicated their readiness with a mouse-click, which was followed with the sequence shown in the figure. After a static viewing period of 1,000 ms, the disks moved along straight lines for 400 ms, deviated by 30–180° clockwise or counter-clockwise, and then moved along straight lines for a further 400 ms. Blue arrows show the directions of motion of the disks and dashed blue lines show their recent trajectories. Dotted lines illustrate one set of potential trajectories of the disks, but were not shown in the actual stimulus. After 800 ms the motion ceased and the observer was cued to report the direction(s) of motion. The cue could query one of the following: post-deviation SR (illustrated in figure), post-deviation FR, pre-deviation SR or pre-deviation FR (see text and demos). The observer reported the direction(s) of motion using a mouse-controlled vector at each disk cued. After all reports were recorded, feedback was provided using red and blue vectors for the actual and reported directions of motion for each cued disk.

Stimuli used to probe events can range in complexity from a few geometric shapes in motion (Zacks et al., 2006) to complex movie scenes (Swallow et al., 2009). Here, in order to probe memory mechanisms underlying events, we used relatively simple stimuli, namely a set of moving disks undergoing a synchronized trajectory-change in mid-course (Figure 2 and demos). This synchronized trajectory-change formed an event boundary clearly splitting the event into two temporally successive pre- and post-deviation segments.

When an event contains multiple segments, observers need to remember not only features and objects, but also which segment these features and objects belong to. In other words, contents of memory need to be “tagged” or associated with segments of events. We consider two alternatives for the operation of SM. According to one model (Figure 1B), which we call the “*Shared Model*”, tagging can take place *both* in SM and STM allowing the brain to use *both* mechanisms simultaneously for different segments of an event. While this has the advantage of providing two types of memory systems to each event segment, its disadvantage is that the complexity of both SM and STM will be high since they will both require tagging operations. The cost of tagging is especially critical for SM compared to STM since it has a much higher capacity than STM and thus requires extensively more tagging operations. That tagging operations take place in SM appear to be supported by a recent study where observers were presented with two temporally successive arrays of letters and were cued to selectively report the first or the second array (Smith et al., 2011). However, as we show in Supplementary Information, the findings of that study can also be explained by an alternative model (Figure 1C) that restricts tagging operations *only* to STM and uses SM as an untagged buffer for the *current* event segment. We call this model the “*Exclusive Model.”* In addition to reducing the complexity for tagging, using SM as an untagged buffer for the current event segment enhances the speed of retrieval. If the only items in SM are from the current event segment, then when a response is needed for an item in the current event segment, there is no need to check tags. This speed advantage is especially critical for SM since its contents decay relatively fast, and any time spent on tagging and tag-checking operations would lead to severe information loss.

The models illustrated in Figures 1B,C can be distinguished by examining the involvement of SM in the recall of information available from the previous event-segment (i.e., before the deviations in the trajectories) and from the current event-segment (i.e., after the deviation in the trajectories). The Shared Model in Figure 1B predicts the involvement of SM for the recall of information related to the previous and the current event-segment, whereas the Exclusive Model in Figure 1C predicts the involvement of SM for the recall of information related to the current event-segment only. To test these sets of predictions we looked for the key signatures for the involvement of SM when recalling information. These signatures are: (i) a decay of recalled information with time; (ii) a better recall of information during single-report, compared to during full-report; and (iii) single-report performance that is comparable to the performance for reporting the first item during full-report. The tables in Figures 1B,C predict the presence (“YES”) and absence (“NO”) of these signatures for the two models.

The experimental paradigm used is a variation of the Multiple Object Tracking (MOT) paradigm (Pylyshyn and Storm, 1988; Tripathy and Barrett, 2003, 2004; Tripathy et al., 2007; Narasimhan et al., 2009; Shooner et al., 2010; Tripathy and Howard, 2012; Öǧmen et al., 2013; Huynh et al., 2015, 2017). The stimuli consisted of a set of moving disks that underwent a synchronized change in trajectories mid-way through their trajectories, yielding two event-segments, referred to as the previous and the current event-segments. Observers reported the direction(s) of motion of the trajectories in each event-segment, in conditions of single-report and full-report. The errors in reporting the direction of motion in the different conditions were transformed to performance measures and these were compared to look for the signatures of SM as listed in the above paragraph. The different experiments varied parameters of the stimuli, such as: duration of motion, trajectory-length, cue-delay and set-size. In each case the role of SM was investigated to distinguish between the two models proposed. A summary of the models can be visualized in the color-coded tables in Figures 1B,C, where green indicates a match and red a mismatch between model predictions and experimental results. The findings largely support the Exclusive Model presented in Figure 1C and the proposition that sensory memory is allocated exclusively to the current event-segment.

## Materials and methods

### Observers

Ten observers participated in the experiments, with some observers participating in more than one experiment. This study was carried out in accordance with the recommendations of University of Bradford Committee for Ethics in Research with written informed consent from all subjects. All subjects gave written informed consent in accordance with the Declaration of Helsinki. They had normal visual acuity. They were highly trained, undergoing a few thousand trials in each task before actual data collection. This ensured that any absence of evidence of use of SM was not simply because performance was poor on account of lack of adequate familiarity with the task. In addition, all observers were younger than 32 years at the time of testing. This ensured that the age-related decline in performance for tracking that kicks in very early in adulthood (Kennedy et al., 2009) did not compromise performance in our observers in the current study.

### Equipment

Experiments used programs written in C++, running on a Dell Precision 670 computer, along with Cambridge Research System's ViSaGe Visual Stimulus Generation hardware. Stimuli were viewed from a distance of 1 m on a 22.5 × 17° CRT Sony FD Trinitron computer with a frame rate of 100 Hz. The visible area of the monitor screen was 39.5 cm (800 pixels) wide and 29.5 cm (600 pixels) high. Each screen pixel subtended 1.694 min of arc in the horizontal and vertical directions. The background luminance of the screen was 64.9 cd/m^2^. Chin-and-forehead rests stabilized the head and the viewing distance. A computer mouse was used to initiate each trial and to report the direction(s) of motion. The room was illuminated with regular fluorescent lighting in order to ensure that the screen-persistence could not be utilized when responding to stimuli.

### Stimuli and procedure

The stimuli consisted of *N* (= 1, 2, 3, or 4) gray disks of diameter 1° moving along random bilinear trajectories at a constant speed, which was typically 5°/s (Figure 2 and demos). The duration of motion was typically 800 ms, but was varied in Experiment 1b. Each trial started with randomly positioned stationary disks presented on the screen. Observers indicated their readiness with a mouse-click, which was followed after a gap of 1 s with the sequence shown in Figure 2. Each disk moved along a straight line, deviated by 30–180° clockwise or counter-clockwise at the mid-point of its trajectory, and then moved along a straight line for the remainder of the trajectory. The deviations of all trajectories occurred synchronously exactly midway through the trajectory, with the deviations for the different trajectories on a trial varying randomly over the above range. The starting points of the trajectories did not overlap and the trajectories were constrained so that they terminated before the disks reached the edge of the display, i.e., the disks did not disappear outside the visible area of the screen, nor did they bounce off the edge of the display. When the motion had ceased the observer was cued to report the direction(s) of motion. To minimize confusion when reporting directions none of the *2N* directions of motion present in each stimulus were within 20° of one another. When trajectories intersected, disks moved across each other without changing direction or speed.

### Reporting conditions

Each trial involved one of four possible reporting conditions (see Figure 2). Observers could be queried on the direction(s) of motion of: a randomly selected pre-deviation trajectory, a randomly selected post-deviation trajectory, all of the pre-deviation trajectories, or all of the post-deviation trajectories. These are referred to as the pre-deviation single report (SR), post-deviation SR, pre-deviation full report (FR), or post-deviation FR conditions. The reporting condition was cued immediately after the termination of stimulus motion as follows:

#### Pre-deviation SR trials

To provide an effective position cue for the pre-deviation segment, each disk disappeared at the end of the trajectory and reappeared at the point of its deviation. The disk marked for report was blue in color, the other disks being gray as before.

#### Post-deviation SR trials

Each disk remained visible and stationary when motion terminated. The disk marked for report turned red.

#### Pre-deviation FR trials

To provide an effective position cue for the pre-deviation segment, each disk disappeared at the end of the trajectory and reappeared at the point of its deviation. All of the disks were marked blue to indicate that they were to be reported.

#### Post-deviation FR trials

All disks remained stationary and turned red to indicate that they were to be reported.

All four reporting conditions were equally likely within a block, which consisted of 10 trials of each condition randomly interleaved. Experiments 1a,b and 3 involved 1600 trials (4 stimulus conditions × 10 blocks × 40 trials/block) for each observer. Experiment 2 with 6 stimulus conditions involved 2400 trials.

When the mouse-cursor was moved close to a marked disk at the end of a trial a pointer appeared linking the disk to the cursor. The direction of the pointer could be adjusted and reported by moving and clicking the mouse. This resulted in the reported disk turning gray. If more items remained to be reported, as in full-report, the reporting cycle was repeated. When all marked items had been reported feedback was provided in the form of color-coded vectors attached to each marked item showing the actual and reported directions of motion. On full-report trials observers were permitted to report the marked items in the order of their choosing and the direction of motions reported and the order of reporting were both recorded.

### Data analysis

Each error, δ, was transformed (Shooner et al., 2010; Öǧmen et al., 2013; Huynh et al., 2015, 2017) using the equation:

$$\begin{array}{l} Transformed\;Performance\;\text{TP}=1-|\delta |/180 \end{array}$$

This linear transformation converts the error into a probability-like number, with perfect performance and chance performance represented by 1.0 and 0.5 respectively. *TP* was averaged separately for each of the 4 reporting conditions for each stimulus condition. For the full-report trials, averages were also obtained for FR1, FR2, FR3, and FR4 (where applicable), the first, second, third and fourth items to be reported, respectively.

Some studies have used RMS error to analyse their results and a question that arises is would our results be any different had we used RMS error instead of TP. We simulated 1,000 trials with an RMS error of 1° and calculated the *TP* for the same 1,000 data. We repeated the simulations for 21 different values of RMS error in the in the range 1–90°. The corresponding values of *TP* ranged from 0.995 to 0.597 and the co-efficient of linear correlation between the RMS errors and their corresponding *TP* values was −0.9999. While it might be theoretically possible to generate non-random data sets where the relationship between the two measures is not that linear, for most data encountered in practical situations, we expect an almost perfect linear correlation between RMS error and *TP*. As for the results of statistical tests conducted, *TP* is a linear transformation of RMS error (and vice versa) and any statistical conclusion reached by analysing data using one measure should be valid if the other measure were to be used instead.

We preferred to use *TP* as a measure of error as its use permits comparison of our data with that from our earlier studies that used the same measure (e.g., Shooner et al., 2010). Additionally the use of TP is intuitively appealing as it is a normalized measure that permits comparisons across experiments and also because of its similarity to probability, with 1.0 and 0.5 representing perfection and chance levels.

### Statistical analysis

Each experiment had 4 or 5 observers, in line with sample sizes used in similar studies (Shooner et al., 2010; Öǧmen et al., 2013; Huynh et al., 2015, 2017). All observers within an experiment participated in all of the experimental conditions for that experiment. Most statistical analyses were performed on *TP* by using RM-ANOVA with significance threshold set to 0.05 (see Table 1). Mauchly's Test was used to check if sphericity assumptions were met. When sphericity assumptions were violated, a Greenhouse-Geisser correction was applied and the correction-factor (ε) is shown in the degrees of freedom column in Table 1.

**Table 1:** Statistical results.

**Statistical test**	**Row number**	**Comparison**	**Experiment**	**Degrees of freedom**	***F*-value**	***p*-value**	***$\eta_{P}^{2}$***	**Shared model (Figure 1B)**	**Exclusive model (Figure 1C)**	
		TP_SR_ > TP_FR_								
2-way Repeated	1	Post-deviation	Exp 1a	1,3	76.683	0.003	0.962	√	√	
Measures ANOVA	2		Exp 1b	1,3	72.519	0.003	0.96	√	√	
	3		Exp 2	1,4	15.963	0.016	0.8	√	√	
	4		Exp 3	1,3	43.284	0.007	0.935	√	√	
	5		Exp3	3	*t* = 5.23	0.013		√	√	SS4
										
	6	Pre-deviation	Exp 1a	1,3	35.118	0.01	0.921	√	**X**	
	7		Exp 1b	1,3	4.002	0.139	0.572	**X**	√	
	8		Exp 2	4	*t* = 0.153	0.886		**X**	√	
	9		Exp 3	1,3	4.535	0.123	0.602	**X**	√	
	10		Exp3	3	*t* = 1.821	0.166		**X**	√	SS4
										
		TP_SR_ = TP_FR1_								
	11	Post-deviation	Exp 1a	1,3	2.661	0.201	0.47	√	√	
	12		Exp 1b	1,3	0.965	0.398	0.243	√	√	
	13		Exp 2	1,4	17.987	0.013	0.818	√	√	
	14		Exp 3	1,3	0.772	0.444	0.205	√	√	
	15		Exp 3	3	*t* = 1.038	0.376		√	√	SS4
										
	16	Pre-deviation	Exp 1a	1,3	29.667	0.012	0.908	**X**	√	
	17		Exp 1b	1,3	16.892	0.026	0.849	**X**	√	
	18		Exp 2	4	*t* = 3.389	0.028		**X**	√	
	19		Exp 3	1,3	10.744	0.047	0.782	**X**	√	
	20		Exp3	3	*t* = 3.453	0.041		**X**	√	SS4
										
		Duration and Cue-delay							
	21	Post-deviation	Exp 1a	3,9	91.411	<0.001	0.968			
	22		Exp 1b	3,9	6.567	0.012	0.686			
	23		Exp 2	5,20	14.418	<0.001	0.783	√	√	
										
	24	Pre-deviation	Exp 1a	3,9	23.603	<0.001	0.887			
	25		Exp 1b	3,9	1.728	0.23	0.366	**X**	√	
										
		Duration								
3-way Repeated	26		Exp 1a	3,9 (ε = 0.411)	107.998	<0.001	0.973			
Measures ANOVA	27		Exp 1b	3,9 (ε = 0.394)	3.077	0.083	0.506			
	28		Exp 3	3,9 (ε = 0.607)	201.348	<0.001	0.985			
										
		EventSegment								
	29		Exp 1a	1,3	64.38	0.004	0.955			
	30		Exp 1b	1,3	80.758	0.003	0.964			
	31		Exp 3	1,3	967.696	<0.001	0.997			
										
		ReportType (SR, FR)								
	32		Exp 1a	1,3	75.189	0.003	0.962			
	33		Exp 1b	1,3	27.866	0.013	0.903			
	34		Exp 3	1,3	20.743	0.02	0.874			
		EventSegment^*^ReportType (SR, FR)							
	35		Exp 1a	1,3	50.145	0.006	0.944	**X**	√	
	36		Exp 1b	1,3	23.677	0.017	0.888	**X**	√	
	37		Exp 3	1,3	47.186	0.006	0.94	**X**	√	
		EventSegment^*^ReportType (SR, FR1)							
	38		Exp 1a	1,3	10.95	0.045	0.785	**X**	√	
	39		Exp 1b	1,3	18.013	0.024	0.857	**X**	√	
	40		Exp 3	1,3	3.863	0.144	0.563	√	**X**	
										
		EventSegment^*^Duration							
	41		Exp 1a	3,9 (ε = 0.358)	1.562	0.299	0.342	**X**	√	
	42		Exp 1b	3,9 (ε = 0.461)	1.703	0.275	0.362	**X**	√	
										
		EventSegment								
2-way Repeated	43		Exp 3	1,3	42.322	0.007	0.934			SS4 (SR,FR)
Measures ANOVA	44		Exp 3	1,3	60.352	0.004	0.953			SS4 (SR,FR1)
		ReportType								
										
	45		Exp 3	1,3	19.505	0.022	0.867			SS4 (SR,FR)
	46		Exp 3	1,3	8.585	0.061	0.741			SS4 (SR,FR1)
										
		EventSegment^*^Report Type							
	47		Exp 3	1,3	21.729	0.019	0.879	**X**	√	SS4 (SR,FR)
	48		Exp 3	1,3	5.849	0.094	0.661	√	**X**	SS4 (SR,FR1)

The rightmost two columns indicate agreement (√) or disagreement (X) with the predictions of the model shown in Figure 1B (“shared model) and in Figure 1C (“exclusive model”); blank entries indicate absence of a strong prediction of the models.

*ε, Greenhouse-Giesser correction factor when assumptions of sphericity were violated; t, t-statistic where ANOVA was inappropriate; SS4, When statistical tests were conducted only on data of set-size = 4*.

### Experimental conditions

In the first experiment, we fixed the cue delay for the post-deviation segment to 0 ms (i.e., the cue appeared immediately at the termination of motion). For the pre-deviation segment, this corresponded to a cue delay equal to half the stimulus duration. To obtain different cue delays for the pre-deviation segment, we varied stimulus duration. In general, with increased cue delay, SM performance decays (Sperling, 1960; Averbach and Coriell, 1961; Coltheart, 1983; Haber, 1983; Breitmeyer and Öǧmen, 2006; Shooner et al., 2010; Öǧmen et al., 2013; Huynh et al., 2015, 2017). In our stimulus, however, since stimulus duration co-varies with cue delay, this decay may be countered by an increase in signal strength through the increase in stimulus duration. To disentangle the two effects, first we examined performance for the post-deviation segment to assess signal strength as a function of duration. For the post-deviation segment, memory is not expected to decay as a function of duration since cue delay is fixed. Thus, any increase in signal strength should be reflected as improved performance as a function of duration. Two conditions were run, one with constant motion speed (Experiment 1a) and one with constant motion trajectory length (Experiment 1b).

#### Constant speed condition (experiment 1a)

Three disks moved along bilinear trajectories at a constant speed of 5°/s for a total duration of 200, 400, 800 or 1200ms. The different durations were tested in different blocks in randomized order.

#### Constant trajectory length condition (experiment 1b)

The three disks moved for the same durations as above, but the speed of the disks co-varied with the duration of motion so that the disks moved through a constant trajectory length of 4°, regardless of the duration or speed of motion.

#### Post-deviation delay experiment (experiment 2)

A delay was introduced between the termination of the motion of the disks and the appearance of the cue (i.e., the change of color of the marked disks) which indicated to the observers which trajectory or trajectories to report. The stimuli were the same as in Experiment 1 except that the stimuli had a fixed duration of 800 ms and on the post-deviation trials there was a cue-delay of 0, 100, 200, 400, 800, or 1,600 ms. This cue-delay on the post-deviation trials was fixed within a block and varied between blocks. The cue on the pre-deviation trials appeared immediately at the termination of motion, corresponding to a cue-delay of 400 ms for the pre-deviation segment.

#### Set-size experiment (experiment 3)

One, two, three, or four disks moved at a speed of 5°/s for 800 ms. The different set-sizes were tested in different blocks in random order.

## Results

**Figure 3:**
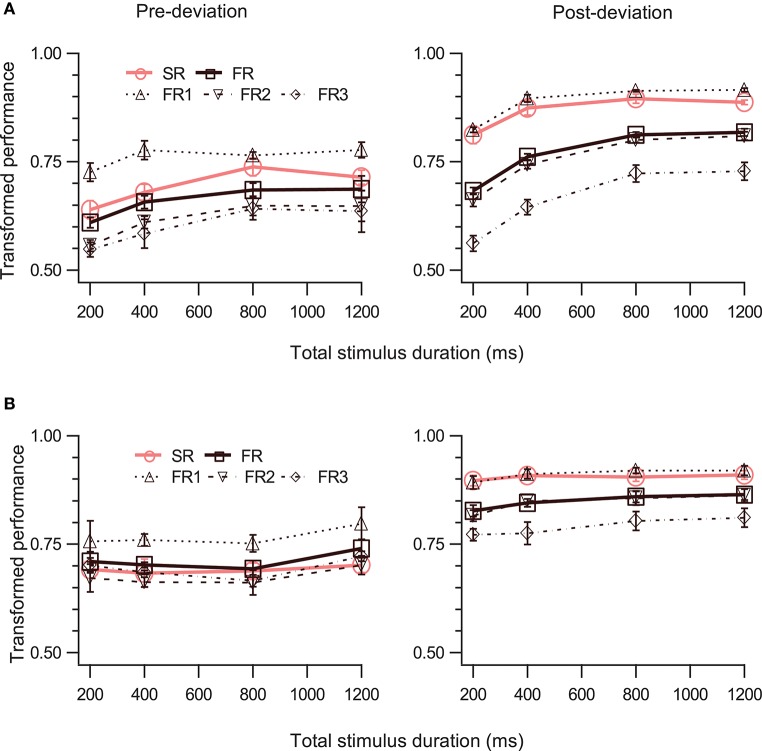
Effect of stimulus duration. The left and right columns show data for pre- and post-deviation conditions, respectively. Transformed performance (TP) averaged across observers (mean ± SEM, *N* = 4) is shown as a function of stimulus duration when the speed of the dots was constant **(A)** and when the trajectory length was constant **(B)**. Each panel shows performance for the SR condition, for the three reports in order of report in the FR condition, and for the FR condition (average of the three reports).

The right panels of Figures 3A,B show performance for the post-deviation trajectory as a function of duration when the speed (Figure 3A) or the trajectory length (Figure 3B) is kept fixed. While there is substantial increase in post-deviation performance as a function of duration when the speed is kept fixed (Figure 3A, right panel; Table 1, row 21), this increase is significantly reduced (from increases of 19.8 and 9.2% to increases of 4.5 and 1.5% for FR and SR respectively relative to the performance for the stimulus with duration of 200 ms), yielding a relatively constant performance when the trajectory length is kept fixed (significant but with a negligible average increase in magnitude of 3.0%: Figure 3B, right panel; Table 1, row 22).

We then examined the pre-deviation segment, in particular for the constant trajectory length case (Figure 3B, left panel), for a decay in performance as a function of cue delay that would indicate the involvement of SM. No such decay can be seen for cue delays ranging from 100 to 600 ms (Table 1, row 25), suggesting that SM is not involved in storing pre-deviation segment information.

Since SM is a rapidly decaying store, single-report performance should be better than full-report performance (TP_SR_ > TP_FR_). Furthermore, since SM is a large capacity store, all items should be stored with approximately equal precision and therefore performance for the single report condition should be approximately equal to the performance for the first item reported in the full-report condition (TP_SR_ = TP_FR1_) (Shooner et al., 2010). A three-way repeated-measures ANOVA (RM-ANOVA) with factors report-type (SR, FR), event-segment (pre- and post-deviation), and duration shows significant main effects of report-type and event-segment (Table 1, rows 30, 33; duration was significant in Expt. 1a but not 1b: rows 26,27) as well as significant two-way interactions between event-segment and report types (Table 1, rows 35, 36, 38, 39) but not between event-segment and duration (Table 1, rows 41,42).

For the first test (TP_SR_ > TP_FR_), a two-way RM-ANOVA shows a significant single-report advantage for the post-deviation trajectory (Table 1, rows 1, 2). On the other hand, for the pre-deviation segment, there was a single-report advantage for the fixed speed condition, where signal strength varied, but this was not significant for the fixed trajectory length condition (Table 1, rows 6,7).

Considering the second SM test (TP_SR_ = TP_FR1_), for the post-deviation segment, TP_SR_ was not significantly different from TP_FR1_ (Table 1, rows 11, 12). In contrast, for the pre-deviation segment, TP_SR_ was significantly different from TP_FR1_ (Table 1, rows 16, 17). Taken together, evidence from this experiment favors strongly the model shown in Figure 1C over the model shown in Figure 1B.

**Figure 4:**
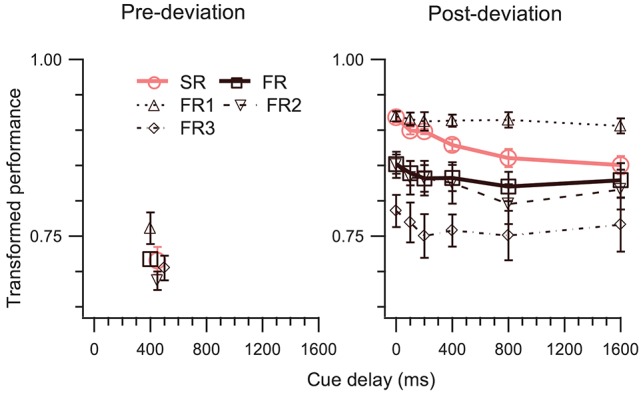
TP (mean ± SEM, *N* = 5) as a function of cue-delay. The left and right panels show data for pre- and post-deviation conditions, respectively. Note that in the pre-deviation conditions the cue-delay was always 400 ms, i.e., the cue was presented as soon as motion of the dots was terminated and 400 ms after the deviation of the trajectories. Some of the symbols in the left panel have been offset by 50 or 100 ms to enhance their visibility.

In Experiment 2, we kept the cue-delay for the pre-deviation segment fixed and varied the cue delay for the post-deviation segment. At a single pre-deviation cue-delay of 400 ms, pre-deviation results (left panel of Figure 4) confirm the findings of Experiment 1, i.e., both SM tests fail (Table 1, rows 8, 18). Results for the post-deviation segment (right panel of Figure 4) support strongly the involvement of SM. Performance decays as a function of cue-delay (Table 1, row 23). TP_SR_ > TP_FR_ is evident at 0 ms cue-delay, but systematically decays so that TP_SR_ approaches TP_FR_ when cue-delay is increased (Table 1, row 3). Similarly, TP_SR_ = TP_FR1_ is evident at 0 ms cue-delay but becomes violated as cue-delay increases (Table 1, row 13). Taken together, results of this experiment also favor the model in Figure 1C over the model in Figure 1B.

**Figure 5:**
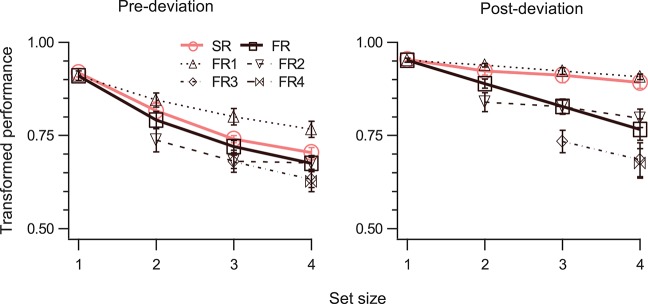
TP (mean ± SEM, *N* = 4) as a function of set-size. The left and right panels show data for pre- and post-deviation conditions, respectively. Each panel shows performance for the SR condition, for up to four reports in order of report in the FR condition, and for the FR condition (average of up to four reports). Note that FR3 and FR4 are not defined when set-size is 2 and FR4 is not defined when set-size is 3.

In general, performance for both SM and STM decreases monotonically as set size increases (Shooner et al., 2010; Öǧmen et al., 2013). However, since STM's capacity is significantly lower than the capacity of SM, this decay is much more pronounced for STM (Öǧmen et al., 2013). To test how performance varies as a function of set-size, in Experiment 3, 1–4 disks moved at a speed of 5°/s for 800 ms and the cue was delivered immediately at the end of motion. The results are shown in Figure 5. A three-way RM-ANOVA shows significant effect of all main factors (Table 1, rows 28, 31, 34), and significant interaction for event-segment and report-type for (SR, FR) but not for (SR, FR_1_) (Table 1, rows 37, 40). TP dropped as the number of disks to be tracked increased, both for pre-deviation and post-deviation trajectories. For post-deviation trajectories, when set-size increased, the drop for FR was steeper than that for SR.

The tests for the difference between SM and STM become more meaningful at larger set-sizes. Therefore, we ran the SM tests for the largest set-size. For the first SM test (TP_SR_ > TP_FR_), a two-way RM-ANOVA showed significant main effects of segment-type, and report-type, and significant interaction (Table 1, rows 43, 45, 47). For the pre-deviation segment TP_SR_ was not significantly different than TP_FR_, while it was for the post-deviation segment (Table 1, rows 5, 10).

For the second SM test (TP_SR_ = TP_FR1_), a two-way RM-ANOVA showed significant main effect of segment-type, marginally significant effect of report-type, but no significant interaction (Table 1, rows 44, 46). Although the interaction was not significant (Table 1, row 48), application of the second test to individual segment-types indicate that TP_SR_ was significantly different from TP_FR1_ for pre-deviation segment, but not for the post-deviation segment (Table 1, rows 15, 20).

## Discussion

The notion of a two-component memory system goes back to nineteenth century. In the 1960s, Atkinson and Shiffrin formalized these components into a model and added control processes for information transfer. Sperling's seminal work (Sperling, 1960) around the same time-period provided the missing interface between the visual inputs and the two-component memory system, namely a sensory memory with high capacity, but of limited duration. Whereas there have been extensive studies on how different stimuli (e.g., letters, digits, shapes, etc.) and stimulus features (e.g., color, orientation, etc.) can be stored in SM, much less is known on how events and event segments are stored in SM. Time is a fundamental dimension for events and motion information can play a critical role in segmenting events (Zacks and Swallow, 2007). Several studies showed that motion information itself can be stored in SM in a way similar to other stimulus properties (Treisman et al., 1975; Demkiw and Michaels, 1976; Blake et al., 1997; Narasimhan et al., 2009; Shooner et al., 2010; Bradley and Pearson, 2012; Öǧmen et al., 2013; Huynh et al., 2015, 2017). Changes in motion information can serve as strong cues for event segmentation (Zacks and Swallow, 2007). Here, we used a simple change in motion information (change of direction) to create an event with two segments and analyzed how SM is allocated to these event-segments. The study was guided by two competing models shown in Figure 1, viz., Shared and Exclusive Models. Inspection of the last two columns of Table 1 shows that, taken together, our results favor overwhelmingly the Exclusive Model (Figure 1C) over the Shared Model (Figure 1B). By using the capacity of SM exclusively for the current event segment, this model eliminates tagging operations so that active vision can operate in real-time.

A question that needs to be addressed is whether the cue at the end of the pre-deviation segment is sufficient for retrieving the appropriate tag. The end point of the pre-deviation segment is also the starting point for the post-deviation segment. Could the cue override this ambiguity? The instructions given to the observers were that they should report the direction of the pre-deviation segment when the color of the cue was blue. However, it is conceivable that the pre-deviation directions were over-written by the post-deviation directions and observers were mis-reporting these in place of the pre-deviation directions. This might explain why the performance was poorer for reporting pre-deviation directions. This is very unlikely, because the large deviation (30–180°, with mean of 105°) between the two segments would produce a mean difference in directions of 75°. If the observers were systematically and accurately reporting the post-deviation direction when asked to report the pre-deviation direction, then the errors would be large and, theoretically, the TP should be no better than 0.583 and simulations suggest that the TP values obtained in the different experimental conditions tested would lie between 0.584 and 0.522, which is well below most of the TP values presented in Figures 3–5. In addition, if the errors were consistently as large as 75°, this would have been very obvious in the visual feedback that was provided at the end of each trial. It is therefore unlikely that the post-deviation directions systematically over-wrote the pre-deviation directions. It is possible that over-writing did occur in a small proportion of the trials and this would have added some noise to the performance curves. However, over-writing of directions is an unlikely explanation for the pattern of results across the different conditions.

An alternative to the Exclusive Model proposed here is that SM simply has no event tagging at all: it simply contains the trajectory information collected over the last few 100 ms or so, without any need for event boundaries. A possible interpretation of the post-deviation results in Figure 3A is that earlier contents of SM were flushed out by later ones as more spatial information was collected. However, this is contradicted by the pre-deviation results in Figure 3B. Suppose SM contains trajectory information collected over 400 ms and flushes out all earlier information. Then when the total stimulus duration is 200 or 400 ms, both pre- and post-deviation information should be available in SM and so these data points in both panels of Figure 3B should show evidence of involvement of SM. While the right panel shows this evidence, the left panel clearly does not (no SR advantage is evident, nor does SR data equal FR1 data, when the stimulus duration is 200 or 400 ms). Similar arguments can be made against collection durations of 800 or 1,200 ms in SM. Therefore this alternate hypothesis that there is no event tagging is not supported by the data, at least for the durations tested in our study.

A further analysis of the data was conducted to rule out the alternate hypothesis that SM simply contains the trajectory information collected over the last few 100 ms and is not influenced by event boundaries. In Experiment 1b the duration of the stimulus was varied, with the briefest duration tested being 200 ms. If event boundaries do not exist, then for this brief stimulus duration, one would expect that the trajectory information in the pre-deviation segments would be integrated with that in the post-deviation segments. Therefore, it would be expected that the error in reporting the direction of the post-deviation segment would be highly correlated with the angle between the pre- and post-deviation segments. For each observer in the 200 ms duration, *single-report* condition in Experiment 1b, we plotted the post-deviation error (in degrees) against the angle (in degrees) between the two segments for the trajectory that was reported for each of the 100 trials. We determined the co-efficient of linear correlation (*r*) between the post-deviation error and the angle between the pre- and post-deviation segments for each set of 100 trials. The co-efficients for the four observers were 0.23, 0.09, 0.36, and 0.24 (respective regression slopes were 0.08, 0.02, 0.11, and 0.04) and the resulting mean of the *r*^2^-values was 0.06. In other words, only about 6% of the variability in the reported post-deviation errors is explained by the direction of the pre-deviation trajectories. This suggests that the trajectory information in pre- and post-deviation segments are not simply integrated; the preferred explanation is that the visual system recognizes that the two segments are to be processed as belonging to separate events.

The definition and segmentation of events may depend not only on stimulus characteristics but also on the goals of the observer (Zacks and Swallow, 2007). The goals of the observer may guide which items are stored in memory. For example, if an observer is looking for a particular set of objects, regardless whether these objects are in the first or second segment of an event, she does not need to attach event-segment tags to items to achieve her goal. Whereas goal-directed flexibility is a signature of STM, future research needs to address to which extent observer's goals can determine whether and how tagging takes place in SM. The fact that observers can resolve the ambiguity in the cue for the pre-deviation trajectories, as discussed above, indicates that goal-directed flexibility may also be a characteristic of SM.

If indeed SM stores exclusively the current event segment, then the question arises as to how SM is reset at the termination of each event segment so as to eliminate all stored items from the prior segment. When event-segmentation is driven by low-level stimulus changes, transients may be used to mask and reset SM (Averbach and Coriell, 1961; Bachmann, 1994; Breitmeyer and Öǧmen, 2006) making it exclusively available to the current event segment. However, a transient signal does not automatically mask and erase all contents of SM: Many factors—such as target-mask spatio-temporal proximity and featural similarity—determine the effectiveness of a transient in erasing the contents of SM (Bachmann, 1994; Breitmeyer and Öǧmen, 2006). In our stimulus, the synchronous deviations in trajectories create a transient signal. This transient signal may be how the contents of SM are erased to allocate SM exclusively to the current event segment. While we have not measured the effectiveness of these transients in masking the preceding motion information, two lines of reasoning suggest that visual transients may be sufficient reset-signals in some contexts but they are not likely to be necessary in setting event boundaries and simultaneously erasing the contents of SM:

First, as mentioned above, spatiotemporal proximity and featural similarity determine the effectiveness of a mask. In our stimulus, post-deviation trajectories are spatially segregated from pre-deviation trajectories and hence they are not likely to be effective masks. The local transients created at the deviation points are not likely to be effective masks for motion trajectories either. This is because of featural dissimilarity (flicker vs linear motion), or because of the fact that pre-deviation motion duration is long and is not likely to be masked effectively with a brief transient.

Second, whereas visual transients are in general associated with event boundaries, events can be segmented without visual transients as well (Zacks and Swallow, 2007). Segments can be created based on cross modal information and/or can be context dependent: For example, a whistle sound coming from a referee in a basketball game may generate a segment boundary whereas, for the identical visual stream, a whistle sound coming from spectators may not generate a segment boundary. Hence, in addition to “traditional” masking mechanisms, we speculate the existence of other mechanisms to control the storage of event information in SM.

Recent research suggests a key role for SM in cognition by showing that it requires attention (Persuh et al., 2012; Öǧmen et al., 2013), the read-out from SM and selective spatial attention share similar neural correlates (Ruff et al., 2007), and SM dynamics correlate with psychological intelligence (Miller et al., 2010) and cognitive impairments (Lu et al., 2005). The structure of the Exclusive Model in Figure 1C dovetails nicely with these findings by showing how SM is allocated dynamically (and possibly with attentional guidance) under ecological viewing conditions.

## Author contributions

ST designed the experiments and collected the data. ST and HÖ analyzed and interpreted the data and wrote the paper. Several undergraduate students also assisted by acting as observers and collating data and performing preliminary analysis of the data and writing reports for parts of the project, as part of their Final Year Research Project module at the School of Optometry and Vision Science, University of Bradford.

### Conflict of interest statement

The authors declare that the research was conducted in the absence of any commercial or financial relationships that could be construed as a potential conflict of interest.

## Supplementary material

Video 1 Simulation of one trial for the post-deviation full-report condition.

Video 2 Simulation of one trial for the post-deviation single-report condition, with a delay between end of stimulus presentation and presentation of the cue.

Video 3 Simulation of one trial for the post-deviation single-report condition.

Video 4 Simulation of one trial for the pre-deviation full-report condition.

Video 5 Simulation of one trial for the pre-deviation single-report condition.

## References

[B1] Alvarez G. A. Cavanagh P. (2004). The capacity of visual short-term memory is set both by visual information load and by number of objects. Psychol. Sci. 15, 106–111. 10.1111/j.0963-7214.2004.01502006.x 14738517

[B2] Atkinson R. C. Shiffrin R. M. (1968). Human memory: a proposed system and its control processes, in The Psychology of Learning and Motivation, Vol. 2, eds Spence K. W. Spence J. T. (New York, NY: Academic Press), 89–195.

[B3] Atkinson R. C. Shiffrin R. M. (1971). The control of short term memory. Sci. Am. 225, 82–90. 10.1038/scientificamerican0871-82 5089457

[B4] Averbach E. Coriell A. (1961). Short-term memory in vision. Bell Syst. Tech. J. 40, 309–328. 10.1002/j.1538-7305.1961.tb03987.x

[B5] Bachmann T. (1994). Psychophysiology of Visual Masking: The Fine Structure of Conscious Experience. New York, NY: Nova Science Publishers, Inc.

[B6] Baddeley A. (2007). Working Memory, Thought, and Action. Oxford, UK: Oxford University Press.

[B7] Baddeley A. D. Hitch G. (1974). Working memory, in The Psychology of Learning and Motivation: Advances in Research and Theory, Vol. 8, ed Bower G. H. (New York, NY: Academic Press), 47–89.

[B8] Bays P. M. Husain M. (2008). Dynamic shifts of limited working memory resources in human vision. Science 321, 851–854. 10.1126/science.1158023 18687968 PMC2532743

[B9] Blake R. Cepeda N. J. Hiris E. (1997). Memory for visual motion. J. Exp. Psychol. Human Percept. Perform. 23, 353–369. 10.1037/0096-1523.23.2.353 9103999

[B10] Bradley C. Pearson J. (2012). The sensory components of high-capacity iconic memory and visual working memory. Front. Psychol. 3:355. 10.3389/fpsyg.2012.00355 23055993 PMC3457081

[B11] Breitmeyer B. G. Öǧmen H. (2006). Visual Masking: Time Slices through Conscious and Unconscious Vision, 2nd Edn. Oxford, UK: Oxford University Press.

[B12] Coltheart M. (1983). Iconic memory. Philos. Trans. R. Soc. B 302, 283–294. 10.1098/rstb.1983.0055 6137847

[B13] Cowan N. (2001). The magical number 4 in short-term memory: a reconsideration of mental storage capacity. Behav. Brain Sci. 24, 87–114. 10.1017/S0140525X01003922 11515286

[B14] Demkiw P. Michaels C. F. (1976). Motion information in iconic memory. Acta Psychol. 40, 257–264. 10.1016/0001-6918(76)90029-9 970200

[B15] Haber R. N. (1983). The impending demise of the icon: a critique of the concept of iconic storage in visual information processing. Behav. Brain Sci. 6, 1–54. 10.1017/S0140525X0001428X

[B16] Huynh D. Tripathy S. P. Bedell H. E. Öǧmen H. (2017). The reference frame for encoding and retention of motion depends on stimulus set size. Atten. Percept. Psychophys. 79, 888–910. 10.3758/s13414-016-1258-5 28092077

[B17] Huynh D. L. Tripathy S. P. Bedell H. E. Öǧmen H. (2015). Stream specificity and asymmetries in feature binding and content-addressable access in visual encoding and memory. J. Vis. 15:14, 10.1167/15.13.14 26382005

[B18] Johansson G. von Hofsten C. Jansson G. (1980). Event perception. Annu. Rev. Psychol. 31, 27–63. 10.1146/annurev.ps.31.020180.000331 7362214

[B19] Kennedy G. J. Tripathy S. P. Barrett B. T. (2009). Early age-related decline in the effective number of trajectories tracked in adult human vision. J. Vis. 9:21, 10.1167/9.2.21 19271931

[B20] Klatzky R. L. (1980). Human Memory: Structures and Processes. New York, NY: Freeman.

[B21] Lu Z. L. Neuse J. Madigan S. Dosher B. A. (2005). Fast decay of iconic memory in observers with mild cognitive impairments. Proc. Natl. Acad. Sci. U.S.A. 102, 1797–1802. 10.1073/pnas.0408402102 15665101 PMC547847

[B22] Makovski T. (2012). Are multiple visual short-term memory storages necessary to explain the retro-cue effect? Psychon. Bull. Rev. 19, 470–476. 10.3758/s13423-012-0235-9 22415524

[B23] Matsukura M. Hollingworth A. (2011). Does visual short-term memory have a high-capacity stage? Psychon. Bull. Rev. 18, 1098–1104. 10.3758/s13423-011-0153-2 21935737 PMC3248760

[B24] Miller R. Rammsayer T. H. Schweizer K. Troche S. J. (2010). Decay of iconic memory traces is related to psychometric intelligence: a fixed-links modeling approach. Learn. Individ. Differ. 20, 699–704. 10.1016/j.lindif.2010.08.010

[B25] Narasimhan S. Tripathy S. P. Barrett B. T. (2009). Loss of positional information when tracking multiple moving dots: the role of visual memory. Vis. Res. 49, 10–27. 10.1016/j.visres.2008.09.023 18930074

[B26] Öǧmen H. (2007). A theory of moving form perception: synergy between masking, perceptual grouping, and motion computation in retinotopic and non-retinotopic representations. Adv. Cogn. Psychol. 3, 67–84. 10.2478/v10053-008-0015-2 20517499 PMC2864981

[B27] Öǧmen H. Ekiz O. Huynh D. Bedell H. E. Tripathy S. P. (2013). Bottlenecks of motion processing during a visual glance: the leaky flask model. PLoS ONE 8:e83671. 10.1371/journal.pone.0083671 24391806 PMC3877086

[B28] Öǧmen H. Herzog M. H. (2010). The geometry of visual perception: retinotopic and non-retinotopic representations in the human visual system. Proc. IEEE Inst. Electr. Electron. Eng. 98, 479–492. 10.1109/JPROC.2009.2039028 22334763 PMC3277856

[B29] Öǧmen H. Herzog M. H. (2016). A new conceptualization of human visual sensory-memory. Front. Psychol. 7:830. 10.3389/fpsyg.2016.00830 27375519 PMC4899472

[B30] Persuh M. Genzer B. Melara R. D. (2012). Iconic memory requires attention. Front. Hum. Neurosci. 6:126, 10.3389/fnhum.2012.00126 22586389 PMC3345872

[B31] Pinto Y. Sligte I. G. Shapiro K. L. Lamme V. A. F. (2013). Fragile visual short-term memory is an object-based and location-specific store. Psychon. Bull. Rev. 20, 732–739. 10.3758/s13423-013-0393-4 23456410

[B32] Pylyshyn Z. W. Storm R. W. (1988). Tracking multiple independent targets: evidence for a parallel tracking mechanism. Spat. Vis. 3, 1–19. 10.1163/156856888X00122 3153671

[B33] Rensink R. A. (2014). Limits to the usability of iconic memory. Front. Psychol. 5:971. 10.3389/fpsyg.2014.00971 25221539 PMC4148905

[B34] Ruff C. C. Kristjánsson A. Driver J. (2007). Readout from iconic memory and selective spatial attention involve similar neural processes. Psychol. Sci. 18, 901–909. 10.1111/j.1467-9280.2007.01998.x 17894608 PMC2440528

[B35] Shooner C. Tripathy S. P. Bedell H. E. Öǧmen H. (2010). High-capacity, transient retention of direction-of-motion information for multiple moving objects. J. Vis. 10:8, 10.1167/10.6.8 20884557 PMC3248821

[B36] Sligte I. G. Scholte H. S. Lamme V. A. F. (2008). Are there multiple visual short-term memory stores? PLoS ONE 3:e0001699. 10.1371/journal.pone.0001699 18301775 PMC2246033

[B37] Smith W. S. Mollon J. D. Bhardwaj R. Smithson H. E. (2011). Is there brief temporal buffering of successive visual inputs? Q. J. Exp. Psychol. 64, 767–791. 10.1080/17470218.2010.511237 21462092

[B38] Sperling G. (1960). The information available in brief visual presentations. Psychol. Monogr. Gen. Appl. 74, 1–29. 10.1037/h0093759

[B39] Swallow K. M. Zacks J. M. Adams R. A. (2009). Event boundaries in perception affect memory encoding and updating. J. Exp. Psychol. Gen. 138, 236–257. 10.1037/a0015631 19397382 PMC2819197

[B40] Treisman A. Russell R. Green J. (1975). Brief visual storage of shape and movement, in Attention and Performance, Vol. 5, eds Rabbitt P. M. A. Dornic S. (London: Academic Press), 699–721.

[B41] Tripathy S. P. Barrett B. T. (2003). Gross misperceptions in the perceived trajectories of moving dots. Perception 32, 1403–1408. 10.1068/p5161 14959800

[B42] Tripathy S. P. Barrett B. T. (2004). Severe loss of positional information when detecting deviations in multiple trajectories. J. Vis. 4, 1020–1043, 10.1167/4.12.4 15669909

[B43] Tripathy S. P. Howard C. J. (2012). Multiple-trajectory tracking. Scholarpedia 7:11287 10.4249/scholarpedia.11287

[B44] Tripathy S. P. Narasimhan S. Barrett B. T. (2007). On the effective number of tracked trajectories in normal human vision. J. Vis. 7:2. 10.1167/7.6.2 17685785

[B45] Van den Berg R. Shin H. Chou W. C. George R. Ma W. J. (2012). Variability in encoding precision accounts for visual short-term memory limitations. Proc. Natl. Acad. Sci. U.S.A. 109, 8780–8785. 10.1073/pnas.1117465109 22582168 PMC3365149

[B46] van Moorselaar D. Olivers C. N. L. Theeuwes J. Lamme V. A. F. Sligte I. G. (2015). Forgotten but not gone: retro-cue costs and benefits in a double-cueing paradigm suggest multiple states in visual short-term memory. J. Exp. Psychol. Learn. Mem. Cogn. 41, 1755–1763. 10.1037/xlm0000124 25867613

[B47] Warren W. H. Shaw R. E. (eds.). (1985). Persistence and Change, in Proceedings of the First International Conference on Event Perception (Mahwah, NJ: Lawrence Erlbaum Associates).

[B48] Zacks J. M. Swallow K. M. (2007). Event segmentation. Curr. Dir. Psychol. Sci. 16, 80–84. 10.1111/j.1467-8721.2007.00480.x 22468032 PMC3314399

[B49] Zacks J. M. Swallow K. M. Vettel J. M. McAvoy M. P. (2006). Visual motion and the neural correlates of event perception. Brain Res. 1076, 150–162. 10.1016/j.brainres.2005.12.122 16473338

[B50] Zhang W. Luck S. J. (2008). Discrete fixed-resolution representations in visual working memory. Nature 453, 233–235. 10.1038/nature06860 18385672 PMC2588137

