# Supplementary material for: Sensory Memory Is Allocated Exclusively to the Current Event-Segment
Source: Front Psychol. 2018 Sep 7;9:1435. doi: 10.3389/fpsyg.2018.01435 (PMC6137426; doi:10.3389/fpsyg.2018.01435)
Supplement: Supplementary file 1 [file Data_Sheet_1.DOCX]

Supplementary Material

Sensory Memory is Allocated Exclusively to the Current Event-Segment

**Srimant P. Tripathy^*^, Haluk Öğmen**

*** Correspondence:** Srimant P. Tripathy: s.p.tripathy@bradford.ac.uk

# Modeling the findings of Smith, *et al.,* (2011)

Smith et al.’s stimuli consisted of an array of 4 digits, followed by a mask, a second array of 4 digits, which in turn was followed by a second mask. After the stimulus presentation, an auditory tone cued the observer to report either the first or the second array (Partial report condition), or both arrays, i.e. all 8 digits (Full report condition). The cue was delivered either immediately after the second mask (0 ms delay with respect to the second mask, corresponding to a delay of 384 ms for the first array of digits and 128 ms for the second array of digits) or 500 ms after the second mask (corresponding to a delay of 884 ms for the first array of digits and 628 ms for the second array of digits). The cue with 0 ms delay could be either full report or partial report. The cue with 500 ms delay was for partial report. Over several experiments, the general trend in their results was that when the results were plotted as a function of cue-delay, partial report performance for the second array showed no or slight decay while partial report performance for the first array showed significant decay approaching or reaching full-report performance. The decay in performance as a function of cue delay for the first array may be interpreted as the involvement of SM for the first segment of the stimulus. On the other hand, this evidence is equivocal since it is not clear why performance does not decay for the second array of digits when the cue is delayed. Here, we will apply the model in Figure 1(c) to show that Smith et al.’s findings can be explained by this model.

To illustrate the idea and produce quantitative results, assume that

1. the capacity of STM is 3 digits,
2. on average, the time to report behaviorally an item is such that, during this time, 1 item is lost from SM (e.g. compare TP for FR1, FR2 and FR3 in Fig. 3 of main article) and 1 item is transferred from SM to STM.

According to the model in Fig. 1(c), at the termination of the second array, SM will contain the four digits from array 2 and STM will contain 3 of the 4 digits from array 1:

STM = {$D_{1}^{1}, D_{2}^{1},D_{3}^{1}$}

SM = {$D_{1}^{2}, D_{2}^{2},D_{3}^{2},D_{4}^{2}$}

where the sets STM and SM represent the contents of each memory and $D_{j}^{i}$ represents j^th^ digit in the i^th^ array.

**A.** The cue delay of 0 ms corresponds to a delay of 128 ms after the offset of the second array. During this time, assume that one of the items is transferred from SM to STM, yielding:

STM = {$D_{1}^{1}, D_{2}^{1},D_{1}^{2}$}

SM = {$D_{2}^{2},D_{3}^{2},D_{4}^{2}$}

1. If the cue is partial report for the first array, the observer will report $D_{1}^{1} \mathrm{and} D_{2}^{1}$ from STM producing a 2/4=**50%** performance.
2. If the cue is partial report for the second array, the observer will start reporting one of the items from SM, say $D_{2}^{2}$. During the reporting of $D_{2}^{2}, one item$will be lost from SM, say $D_{4}^{2}$ and one item, $D_{3}^{2},$ will be transferred from SM to STM.

After the first report, we will have

STM = {$D_{1}^{1},D_{1}^{2}$,$D_{3}^{2}$}

SM = {}

Following the first report^[[1]](#footnote-1)^, the observer will report the $D_{1}^{2}$and $D_{3}^{2},$ from STM. This will produce a performance of 3/4=**75%**.

The partial report composite performance will be (.5+.75)/2=**62.5%.**

1. If the cue is for full report, the situation will be similar to partial report for the second array; the observer will also report $D_{1}^{1}$ yielding a full report performance of 4/8=**50%**.

**B.** The cue delay of 500 ms corresponds to a delay of 628 ms after the offset of the second array. During this time, assume that two items from array 2 are transferred from SM to STM, and one item is lost in SM, yielding:

STM = {$D_{1}^{1}, D_{1}^{2},D_{2}^{2}$}

SM = {$D_{3}^{2}$}

1. If the cue is partial report for the first array, report $D_{1}^{1}$ from STM, yielding a performance of 1/4=**25%.**
2. If the cue is partial report for the second array, the observer will report $D_{3}^{2}$ from SM and $D_{1}^{2},D_{2}^{2}$ from STM, yielding a performance of 3/4=**75%.**

The partial report composite performance will be (.25+.75)/2=**50%.**

These results are plotted below following the same format as the data in Smith et al. (2011).

**Figure S0:** Plot of predicted performance according the analysis of model in Fig. 1C, following the plotting convention used by Smith, *et al*., (2011).

While the analysis presented above is relatively simplistic, it captures the main findings from Smith, *et al*., (2011), namely how the report for the first array may decay without any substantial decay for the second array.

# Data for individual observers

The following figures show data for individual observers for Experiments 1a (Fig. S1), 1b (Fig. S2), 2 (Fig. S3) and 3 (Fig. S4). As before, the data for pre-deviation trajectories are shown on the panels on the left and for post-deviation trajectories on the panels on the right.

**Figure S1.** Data for individual observers for Experiment 1a. The format is similar to that of Figure 3a, but each row represents data for an individual observer. The error bars showing S.E.s are no longer appropriate since n=1, and are not shown in this and subsequent figures.

**Figure S2.** Data for individual observers for Experiment 1b. The format is similar to that of Figure 3b, but each row represents data for an individual observer.

**Figure S3.** Data for individual observers for Experiment 2. The format is similar to that of Figure 4, but each row represents data for an individual observer.

**Figure S4.** Data for individual observers for Experiment 3. The format is similar to that of Figure 5, but each row represents data for an individual observer.

1. Note that the reported item from SM (and the item transferred from SM to STM, (are not listed in SM anymore because leaving them there won’t affect performance calculations: is already reported and, due to the rapid decay in SM, the representation of STM will be superior to that in SM.


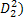

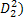

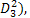

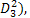

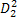

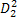

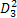

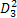


   [↑](#footnote-ref-1)
